# Switch to Stribild versus continuation of NVP or RPV with FTC and TDF in virologically suppressed HIV adults: a STRATEGY-NNRTI subgroup analysis

**DOI:** 10.7448/IAS.17.4.19793

**Published:** 2014-11-02

**Authors:** Hans-Juergen Stellbrink, Andrea Antinori, Anton Pozniak, Jason Flamm, Fritz Bredeek, Kiran Patel, Will Garner, David Piontkowsky

**Affiliations:** 1 Infectious Diseases, ICH Study Center, Hamburg, Germany; 2 Infectious Diseases, Istituto Nazionale Malattie Infettive Lazzaro Spallanzani IRCCS, Rome, Italy; 3 HIV Unit, St Stephens Centre, Chelsea and Westminster Hospital, London, UK; 4 Infectious Diseases, Kaiser Permanente, Sacramento, CA, USA; 5 HIV Medicine, Metropolis Medical Group, San Francisco, CA, USA; 6 HIV Division, Gilead Sciences, Foster City, CA, USA

## Abstract

**Introduction:**

Switch to Stribild (STB) was non-inferior to continuation of a non-nucleoside reverse transcriptase inhibitor (NNRTI) with emtricitabine and tenofovir DF (FTC/TDF) at week 48 in virologically suppressed HIV adults [1]. We report the Week 48 efficacy and safety of STB versus nevirapine (NVP) or rilpivirine (RPV) with FTC/TDF in suppressed subjects.

**Materials and Methods:**

Virologically suppressed subjects on an NNRTI with FTC/TDF regimens for ≥6 months were randomized (2:1) to switch to STB versus continue their NNRTI regimen. Eligibility criteria included no documented resistance to FTC and TDF, no history of virologic failure and eGFR ≥70 mL/min. The primary endpoint was the proportion of subjects in the modified ITT population who maintained HIV-1 RNA <50 copies(c)/mL at Week 48 by FDA snapshot algorithm (12% non-inferiority margin). Subgroup analysis by non-EFV NNRTI use (NVP [74]; RPV [19]; etravirine [3]) at screening was pre-specified.

**Results:**

The mITT population included 433 subjects who were randomized and treated. In the non-EFV NNRTI subgroup, 59 switched to STB; 37 continued a non-EFV NNRTI (27 NVP, 10 RPV) with FTC/TDF. At week 48, 97% STB versus 95% non-EFV NNRTI maintained HIV-1 RNA <50 c/mL. No emergent resistance was detected in either group. No difference in median increases from baseline in CD4 count at week 48 (cells/µL): 25 STB versus 55 non-EFV NNRTI (p=0.78). No discontinuation due to adverse events; no cases of proximal renal tubulopathy. As expected, there were no significant changes in the frequency of neuropsychiatric symptoms (i.e. anxiety, insomnia, dizziness, vivid dreams, weird/intense dreams, and nightmares) reported on the HIV Symptom Index at week 48 compared to baseline after switching to STB. There was a greater but non-progressive decrease from baseline in eGFR in the STB versus non-EFV NNRTI group; median changes (mL/min) at week 48: −9.1 versus −1.4. Switch to STB was associated with a higher treatment ease (convenience, flexibility, demand, lifestyle, understanding) score (range: −15 to 15) at week 4 (median: 14 vs 11; p=0.047) and week 24 (median: 14 vs 12.5; p=0.038).

**Conclusions:**

In this small group of virologically suppressed subjects, switch to STB vs continuation of NVP or RPV with FTC/TDF was safe, well-tolerated, and associated with a high rate of virologic suppression at week 48. There was more treatment ease with STB use.

## References

[1] Pozniak A, Markowitz M, Mills A, Stellbrink HJ, Antela A, Domingo P (2014). Switching to coformulated elvitegravir, cobicistat, emtricitabine, and tenofovir versus continuation of non-nucleoside reverse transcriptase inhibitor with emtricitabine and tenofovir in virologically suppressed adults with HIV (STRATEGY-NNRTI): 48 week results of a randomised, open-label, phase 3b non-inferiority trial. Lancet Infect Dis.

